# Phospholipid Derivatives of Cinnamic Acid Restore Insulin Sensitivity in Insulin Resistance in 3T3-L1 Adipocytes

**DOI:** 10.3390/nu13103619

**Published:** 2021-10-15

**Authors:** Małgorzata Małodobra-Mazur, Dominika Lewoń, Aneta Cierzniak, Marta Okulus, Anna Gliszczyńska

**Affiliations:** 1 Department of Forensic Medicine, Division of Molecular Techniques, Wroclaw Medical University, Sklodowskiej-Curie 52, 50-369 Wrocław, Poland; dominikalewon@gmail.com (D.L.); aneta.cierzniak@student.umw.edu.pl (A.C.); 2 Department of Chemistry, Wrocław University of Environmental and Life Sciences, Norwida 25, 50-375 Wrocław, Poland; marta.b.czarnecka@gmail.com

**Keywords:** insulin resistance, insulin signaling, cinnamic acid, 3-methoxycinnamic acid, phosphatidylcholine, conjugates

## Abstract

Background: Insulin resistance (IR) is a condition in which the physiological amount of insulin is insufficient to evoke a proper response of the cell, that is, glucose utilization. Metformin is the first choice for therapy, thanks to its glycemic efficacy and general tolerability. In addition, various natural compounds from plant extracts, spices, and essential oils have been shown to provide health benefits regarding insulin sensitivity. In the present study, we analyzed the effect of phospholipid derivatives of selected natural aromatic acids on insulin action and their potential use to overcome insulin resistance. Methods: The 3T3-L1 fibroblasts were differentiated into mature adipocytes; next, insulin resistance was induced by palmitic acid (16:0). Cells were further cultured with phenophospholipids at appropriate concentrations. To assess insulin sensitivity, we measured the insulin-stimulated glucose uptake, using a glucose uptake test. Results: We showed that cinnamic acid (CA) and 3-methoxycinnamic acid (3-OMe-CA) restored the proper insulin response. However, 1,2-dicinnamoyl-*sn*-glycero-3-phosphocholine (1,2-diCA-PC) and 1-cinnamoyl-2-palmitoyl-*sn*-glycero-3-phosphocholine (1-CA-2-PA-PC) improved insulin sensitivity in insulin-resistant adipocytes even stronger, exhibiting more beneficial effects. Conclusions: The binding of aromatic acids to phosphatidylcholine increases their beneficial effect on insulin sensitivity in adipocytes and expands their potential practical application as nutraceutical health-promoting agents.

## 1. Introduction

Insulin resistance (IR) is the condition in which the physiological amount of insulin is not sufficient to evoke a proper response of the cell, that is, glucose utilization [1]. For that reason, glucose utilization is delayed. Therefore, as a result, elevated glucose levels persist much longer after a meal. Moreover, increased glucose level further stimulates the insulin secretion from pancreatic β-cells, resulting in peripheral hyperinsulinemia. Both hyperglycemia and hyperinsulinemia are conditions leading to the development of type 2 diabetes [2]. Insulin resistance and other metabolic disorders known as metabolic syndrome constitute a significant health problem all over the world, affecting both adults and children, mainly in highly developed countries. They are also rapidly growing problems in medium-urbanized and economically developing countries [3,4,5,6]. There are indeed slight differences in the number of diagnosed cases depending on the country, although metabolic disorders are still a significant health problem and, thus, an economic problem as well.

The main factors leading to insulin resistance development are excess calorie intake, sedentary lifestyle, and lack of physical activity. Genetic background is also taken into account, predisposing to metabolic disorders [7] including epigenetic modifications [8,9]. In addition, insulin resistance is reported as a physiological aging process [10] as well as in other pathological conditions such as polycystic ovary syndrome [11], chronic kidney failure [12], or heart failure [13]. Additionally, insulin resistance might be developed during some pharmacological therapy, mainly glucocorticoids [14], anti-HIV drugs [15], or statins treatment [16].

Numerous diagnostic methods are available, allowing for insulin resistance diagnosis. The most commonly used test to assess insulin sensitivity is the oral glucose tolerance test (OGTT) with subsequent glucose and insulin concentration measurements before glucose administration and after (1 h and 2 h time points). However, some practitioners recommend more informative tests, such as euglycemic hyperinsulinemia clamp (EHC) or insulin sensitivity test (IST) that is considered the gold-standard method to assess the carbohydrates metabolism and overall insulin sensitivity [17].

In the early stages of insulin resistance pathogenesis, patients are often asymptotic (without polyuria, polydipsia, or unintentional weight loss). The patient often becomes aware of being resistant to insulin when developing full-blown type 2 diabetes. Treatment of such patients with developed type 2 diabetes mellitus at the very first step generally includes diet and lifestyle education to achieve normoglycemia via weight reduction, diet and exercise, evaluation for vascular or cardiovascular complications, and the minimization of other long-term risk factors [18]. Initial pharmacological treatment generally begins with metformin [19] or drugs belonging to thiazolidinediones (rosiglitazone, pioglitazone), which act by abolishing insulin resistance, while favorably affecting lipid metabolism. Metformin is the first choice of therapy, thanks to its glycemic efficacy and general tolerability. Furthermore, in contrast to thiazolidinediones, it does not cause weight gain and is not associated with the risk of cardiovascular diseases [20]. The choice of additional therapeutics should be individualized and take into account their cardiovascular effect, renal function, efficacy, side effects, weight effects, risk of hypoglycemia, price, and patient preferences.

Except for the pharmacological agents, various natural compounds derived from plant extracts, spices, herbs, and essential oils have been demonstrated to provide health benefits concerning insulin sensitivity, while being distributed in combination with pharmacotherapy or as the replacement therapy in the early stages of the disease [20,21]. Regarding the use of plants in the treatment of diabetes, it is well known that those traditionally implemented in this chronic disease are rich sources of phenolic acids. Phenolic acids are a class of widely distributed phenolic compounds that are considered important biologically active agents that prevent the development of type 2 diabetes and its complications or other metabolic disorders such as obesity, lipids disorders, or insulin resistance [22,23]. It also represents promising cancer therapeutics, affecting both cancer cell growth and proliferation [24].

The positive impact of that group of natural compounds has been confirmed by numerous studies on animal models showing that phenolic acids added to the diet limit the development of many diseases, including diabetes. Especially well known as an antidiabetic agent in this group is cinnamic acid (CA) and its methoxy derivatives. Administration of cinnamic acid to diabetic rats at doses of 5 and 10 mg/kg of BW improved glucose tolerance in a dose-dependent manner. The results obtained from the administration of 10 mg/kg cinnamic acid were comparable to that of glibenclamide (5 mg/kg) used as a standard. Hafizur et al. also reported that CA significantly improved glucose-stimulated insulin secretion in isolated islets [23]. Caffeic acid and isoferulic acid, when administrated intravenously to streptozotocin-treated rats, reduce the fasting glycemia and attenuate the increase in plasma glucose in an intravenous glucose tolerance test [23,24,25]; whereas, for 4-methoxycinnamic acid, insulinotropic and antihyperglycemic properties were determined [26,27,28,29].

**Figure 1:**
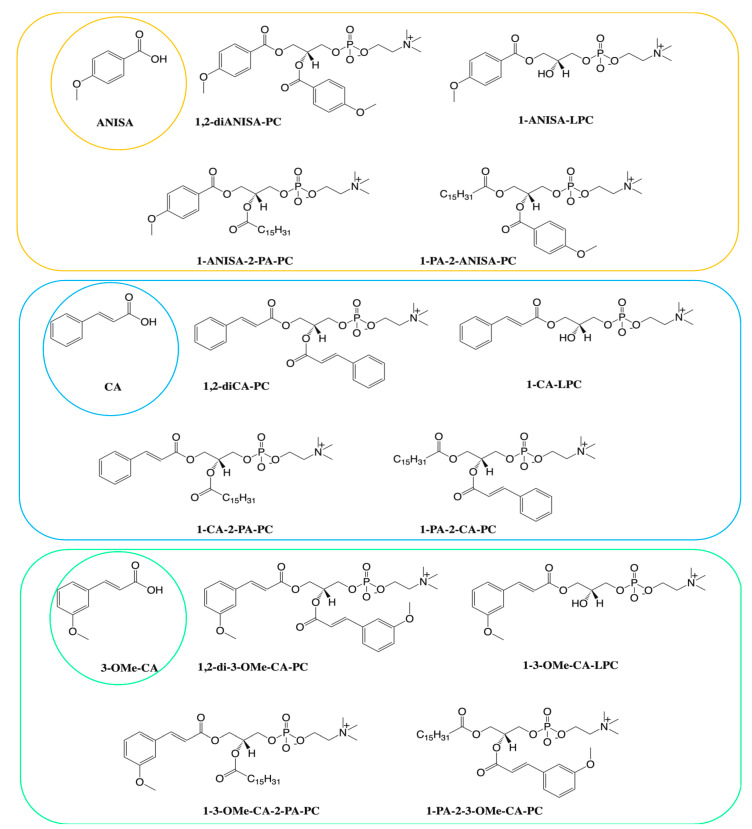
Studied conjugates of phospholipids and aromatic acids: *p*-anisic acid (ANISA), 1,2-dianisoyl-*sn*-glycero-3-phosphocholine (1-diANISA-PC), 1-palmitoyl-2-anisoyl-*sn*-glycero-3-phosphocholine (1-PA-2-ANISA-PC), 1-anisoyl-2-palmitoyl-*sn*-glycero-3-phosphocholine (1-ANISA-2-PA-PC), cinnamic acid (CA), 1,2-dicinnamoyl-*sn*-glycero-3-phosphocholine (1,2-diCA-PC), 1-palmitoyl-2-cinnamoyl-*sn*-glycero-3-phosphocholine (1-PA-2-CA-PC), 1-cinnamoyl-2-palmitoyl-*sn*-glycero-3-phosphocholine (1-CA-2-PA-PC), 3-methoxycinnamic acid (CA), 1,2-di(3-methoxycinnamoyl)-*sn*-glycero-3-phosphocholine (1,2-di-3-OMe-CA-PC), 1-palmitoyl-2-(3-methoxycinnamoyl)-*sn*-glycero-3-phosphocholine (1-PA-2-3OMe-CA-PC), 1-(3-methoxycinnamoyl)-2-palmitoyl-*sn*-glycero-3-phosphocholine (1-CA-2-PA-PC).

The limitations of cinnamic acid and methyl cinnamate are difficulties to achieving in practice a pro-health impact in vivo. Most of the naturally occurring phenolic acids are present in a bounded form, the other aspect is quite fast elimination from the organism, mainly by urine and bile. Due to the fast metabolism and low bioavailability of phenolic acids, we synthesized the series of phospholipid derivatives of selected natural aromatic acids being the derivatives of cinnamic and benzoic acids (Figure 1). For the conjugates in the form of lysophosphatidylcholines, we confirmed that they are able to induce glucose-stimulated insulin secretion (GSIS) and intracellular calcium flux [24,30,31].

Since our previous studies afforded us to discuss mainly the aspect of phenolic acids and the impact of their phospholipid derivatives on insulin secretion from pancreatic β-cells, here, we present the effect of phospholipid derivatives of anisic (ANISA), cinnamic (CA), and 3-methoxycinnamic (3-OMe-CA) acids (Figure 1) on insulin action and their potential usage in overcoming insulin resistance, as exemplified by the murine adipocyte cell line.

## 2. Materials and Methods

### 2.1. Chemicals and Reagents

Phosphatidylcholines containing in the structure the aromatic acids: 1,2-dianisoyl-*sn*-glycero-3-phosphocholine (1-diANISA-PC), 1-palmitoyl-2-anisoyl-*sn*-glycero-3-phosphocholine (1-PA-2-ANISA-PC), 1-anisoyl-2-palmitoyl-*sn*-glycero-3-phosphocholine (1-ANISA-2-PA-PC), 1,2-dicinnamoyl-*sn*-glycero-3-phosphocholine (1,2-diCA-PC), 1-palmitoyl-2-cinnamoyl-*sn*-glycero-3-phosphocholine (1-PA-2-CA-PC), 1-cinnamoyl-2-palmitoyl-*sn*-glycero-3-phosphocholine (1-CA-2-PA-PC), 1,2-di(3-methoxycinnamoyl)-*sn*-glycero-3-phosphocholine (1,2-di-3-OMe-CA-PC), 1-palmitoyl-2-(3-methoxycinnamoyl)-*sn*-glycero-3-phosphocholine (1-PA-2-3OMe-CA-PC), 1-(3-methoxycinnamoyl)-2-palmitoyl-*sn*-glycero-3-phosphocholine (1-CA-2-PA-PC) were synthesized in the Department of Chemistry of Wrocław University of Environmental and Life Sciences, according to the previously reported procedure [24,30]. The free anisic acid (ANISA), cinnamic acids (CA), and 3-methoxycinnamic acids (3-OMe-CA) were purchased from Sigma Aldrich. The stock solutions were prepared from synthesized compounds by solubilizing them in the mixture of organic solvents ethanol: DMSO (*v/v* 1:1) and were later diluted in cell culture media.

### 2.2. 3L3-L1 Culturing and Differentiation

Fibroblasts of 3T3-L1 (purchased from ATCC (Manassas, VA, USA), CL-173™) were cultured in a DMEM (Dulbecco’s Modified Eagle’s Medium, Corning Incorporated, New York, NY, USA), supplemented with 10% fetal calf serum (FCS, Sigma-Aldrich, Saint Louis, MI, USA) and antibiotics (penicillin, 50 U/mL; streptomycin, 50 µg/mL, Corning Incorporated, New York, NY, USA), in a humidified incubator at 37 °C and 5% CO_2_ to achieve 100% confluence. Differentiation to mature adipocytes was induced by culturing 3T3-L1 in DMEM medium containing 10% fetal bovine serum (FBS), antibiotics (penicillin, 50 U/mL; streptomycin, 50 µg/mL), 3-isobutyl-1-methylxanthine (115 µg/mL), dexamethasone (390 ng/mL), and insulin (10 µg/mL) for three days. Next, the medium was changed to DMEM with antibiotics, 10% FBS, and insulin (10 µg/mL). After three more days, the medium was changed to DMEM with antibiotics and 10% FBS, and cells were further cultured for additional two days. The 3T3-L1 fibroblasts differentiated into mature adipocytes after eight days after the initiation of differentiation.

### 2.3. Viablitily Test (MTT)

The viability test was carried out using an MTT (3-(4,5-dimethylthiazol-2-yl)-2,5-diphenyltetrazolium bromide, a tetrazole, Sigma-Aldrich) assay to assess the maximum concentration of analyzed compounds that did not affect the cell viability. First, the cells were incubated for 24 and 48 h in a medium containing phospholipid derivatives over a wide range of concentrations (25–450 µM). For the control cells, ethanol or DMSO was used. Next, the MTT solution in the concentration of 5 mg/mL was added, and cells were further cultured for 3.5 h at 37 °C. The medium with MTT was removed, wells were washed carefully with PBS (ITD, PAN, Wroclaw, Poland), and the purple formazan was dissolved in DMSO (Corning Incorporated). Absorbance was measured at 590 nm with a reference filter of 620 nm. The viability was assessed based on three independent experiments.

### 2.4. Insulin Resistance Induction and the Effect of Phospholipid Derivatives

Insulin resistance was induced in mature 3T3-L adipocytes at the end of adipogenesis by adding palmitic acid (16:0, Sigma-Aldrich) to the final concentration of 0.5 mM. The 3T3-L1 adipocytes were cultured with palmitic acid (16:0) for 48 h, resulting in insulin resistance [32]. Next, phospholipid derivatives in appropriate concentrations were added for a further period of 48 h in order to check if the compounds were able to abolish the developed insulin resistance. Phospholipid derivatives were added to the 3T3-L1 adipocytes along with palmitic acid (16:0), which maintained insulin resistance. The insulin resistance induction in 3T3-L1 adipocytes was assessed based on three independent experiments.

### 2.5. Glucose Uptake Test

The glucose uptake test was carried out to assess the amount of insulin-stimulated glucose uptake. The Glucose Uptake-Glo Assay (Promega Corporation, Madison, WI, USA) was performed after two days of incubation with phospholipid derivatives. The day before the glucose uptake test, cells were starved in a serum-free culture medium overnight. Just before starting the experiment, a cultured medium was discarded, and cells were washed twice with PBS to remove the remaining glucose. Next, part of the experimental cells was stimulated with 1 µM insulin in PBS for 10 min, following 10 min incubation with 10 mM of 2-deoxyglucose (2DG6P). Next, cells were subjected to further processing according to the manufacturer’s protocol. The luminescent was measured using Victor3, 1420 Multilabel Plate Reader, PerkinElmer. Briefly, the 2-deoxyglucose (2DG) was transported across the membrane to the cell in an insulin-dependent manner; however, enzymes could not further modify 2DG6P and accumulated in the adipocytes. After a brief period of incubation, an acid detergent solution (Stop Buffer) was added causing cell lysis. Next, neutralization buffer was added to neutralize the acid, followed by a detection reagent. G6PDH oxidized 2DG6P to 6-phosphodeoxygluconate and simultaneously reduced NADP+ to NADPH. The NADPH was, then, used by reductase to convert the proluciferin to luciferin, which was, then, used to produce a luminescent signal that was proportional to the concentration of 2DG6P. The glucose uptake in experimental and control cells was assessed based on three independent experiments.

### 2.6. Statistical Analysis

Statistical analyses were performed using Statistica13.1 (StatSoft, Tulsa, OK, USA). The normality of the variable distribution was checked using the Shapiro–Wilkes test. For analysis of differences between groups, Student’s *t*-test was used. The statistical significance was set at *p* < 0.05.

## 3. Results

### 3.1. Viability Tests

The viability test of 3T3L1 cells was carried out in a wide range of compound concentrations starting from 25 µM up to 450 µM for all analyzed compounds. As the controls, ethanol or DMSO was used, depending on the type of dissolvent.

**Figure 2:**
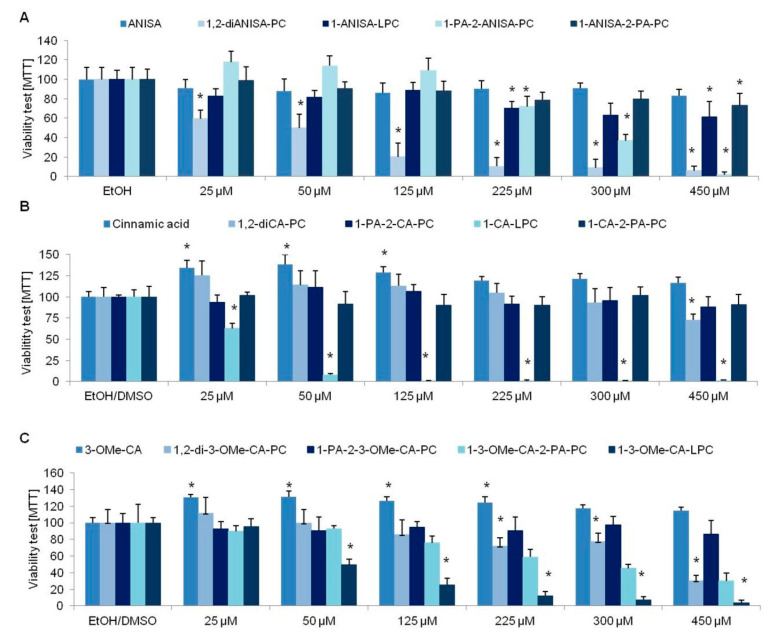
The effect of various concentrations of phospholipid derivatives of anisic acid (**A**), cinnamic acid (**B**), and 3-methoxycinnamic acid (**C**) on the viability of 3T3-L1 cells. Student’s *t*-test, * *p* < 0.05.

Anisic acid did not influence the viability of cells at any of the analyzed concentrations, similar to the following derivatives: 1-ANISA-LPC and 1-ANISA-2-PA-PC. However, we noticed reduced viability of cells cultured with the following derivatives of anisic acid: 1,2-diANISA-PC and 1-PA-2-ANISA-PC (Figure 2A). Cells cultured with 1,2-diANISA-PC showed a reduced rate of viability even at 25 µM. For the second derivative of anisic acid 1-PA-2-ANISA-PC, the reduction in the viability of 3T3-L1 cells was observed at the concentration of 225 µM.

Similar to anisic acid, cinnamic acid did not influence the viability of the tested cells. We even showed the increased rate of mitochondrial metabolism at all analyzed concentrations (Figure 2B). A similar observation was noticed for 3-OMe-CA acid. The following derivatives of cinnamic acid that did not show any influence on the viability of cells are 1,2-diCA-PC, 1-PA-2-CA-PC, 1-CA-2-PA-PC, and 1-PA-2-3-OMe-CA-PC. However, the following cinnamic acid derivatives were shown to reduce the 3T3-L1 viability: 1-CA-LPC, 1,2-Di3-OMe-CA-PC, 1-3OMeCA-LPC, and 1-3-OMeCA-2-PA-PC (Figure 2B,C).

**Figure 3:**
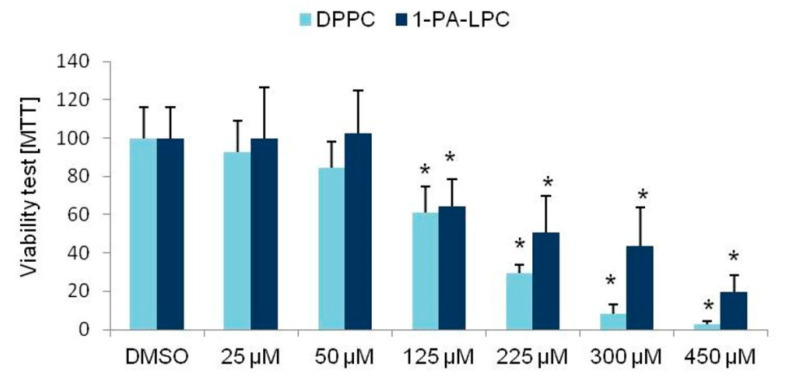
The 3T3-L1 cells viability with the addition of various concentrations of the compounds used as functional group donors used for the synthesis of the phospholipid derivatives of anisic and cinnamic acids. Student’s *t*-test, * *p* < 0.05.

We also checked the viability of the cells cultured with the addition of functional groups, that is, DPPC (dipalmitoylphosphatidylcholine) and 1-PA-LPC. The analyzed compounds influenced the viability of the studied cells at the concentration of 125 µM (Figure 3).

### 3.2. Insulin Resistance Induction and the Glucose Uptake

**Figure 4:**
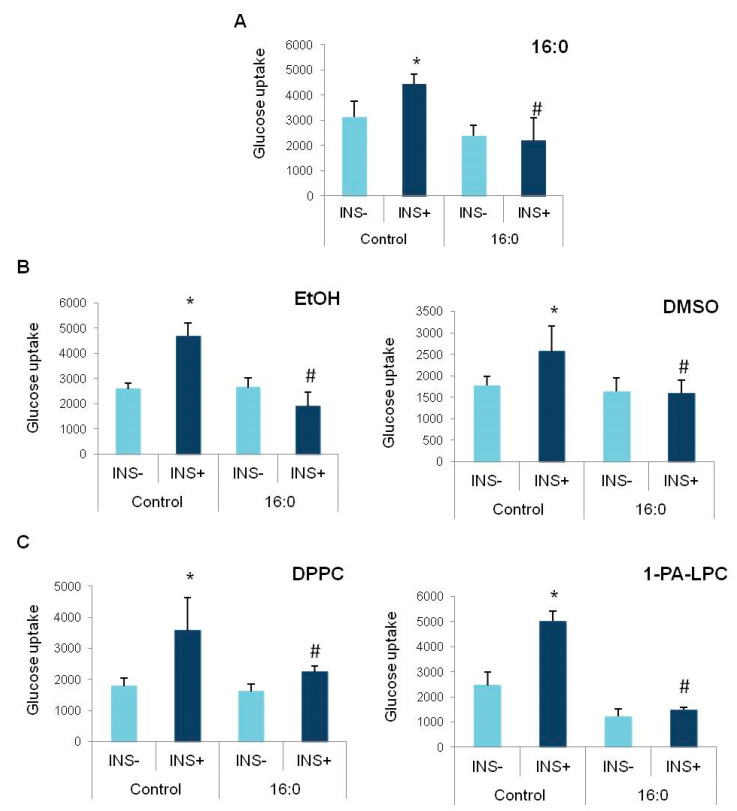
The insulin-stimulated glucose uptake after insulin resistance induction in 3T3-L1 adipocytes. (**A**) The confirmation of insulin resistance induction by palmitic acid (16:0), (**B**) the effect of solvents (ethanol and DMSO) on insulin sensitivity in control and insulin-resistant 3T3-L1 adipocytes, (**C**) the effect of functional compounds used for the synthesis of derivatives (DPPC and 1-PA-LPC) on insulin sensitivity in insulin-sensitive (control) and insulin-resistant (16:0) 3T3-L1 adipocytes, * statistical significance in relation to basal glucose uptake (INS−), *p* < 0.05; # statistical significance in relation to insulin-sensitive cells stimulated by insulin (Control INS+); Student’s *t*-test, *p* < 0.05.

Insulin resistance was induced in 3T3-L1 adipocytes at the fully mature stage (D8 of differentiation) by 0.5 mM of palmitic acid (16:0). After 48 h, the 3T3-L1 adipocytes were resistant to insulin; insulin did not increase the glucose uptake rate in adipocytes treated with palmitic acid (16:0), contrary to control, insulin-sensitive adipocytes, not treated with palmitic acid (16:0) (*p* < 0.000, Figure 4A), which proved the insulin-resistant stage that was developed in the experimental cells.

Furthermore, we also checked whether the solvents themselves influence the glucose uptake by insulin-resistant adipocytes. For that reason, after insulin resistance induction, cells were further cultured either in ethanol or DMSO for the additional period of 48 h. We did not detect any influence of ethanol or DMSO on the insulin-stimulated glucose uptake by insulin-resistant adipocytes. The glucose uptake rate was much lower in insulin-resistant adipocytes, comparing to the control cells with the proper insulin response (EtOH *p* < 0.000, DMSO *p* = 0.010, Figure 4B). On the other hand, none of the solvents used in the study induced insulin resistance in control adipocytes. The glucose uptake after insulin stimulation properly increased in control cells treated with an appropriate solvent (EtOH *p* = 0.006, DMSO *p* = 0.037, Figure 4B).

Similarly, we analyzed if the functional group donors themselves influence the glucose uptake rate. After insulin resistance induction in experimental cells, followed by further incubation with DPPC and 1-PA-LPC, the insulin-stimulated glucose uptake rate was measured. Glucose uptake after insulin stimulation in insulin-resistant adipocytes (16:0, INS+) treated with DPPC or 1-PA-LPC was similar to basal glucose uptake of insulin resistant adipocytes, which indicates that these compounds do not improve insulin sensitivity themselves (DPPC *p* = 0.005; 1-PA-LPC *p* < 0.000, Figure 4C). Furthermore, both DPPC and 1-PA-LPC did not inhibit the insulin-stimulated glucose uptake in control adipocytes with proper insulin sensitivity. The increase after insulin stimulation was significant for both of the analyzed compounds, comparing to basal glucose uptake (INS−) (DPPC *p* = 0.002; 1-PA-LPC *p* = 0.008, Figure 4C).

### 3.3. The Influence of Anisic Acid and Its Phospholipid Derivatives on Glucose Uptake in Insulin-Resistant Adipocytes

**Figure 5:**
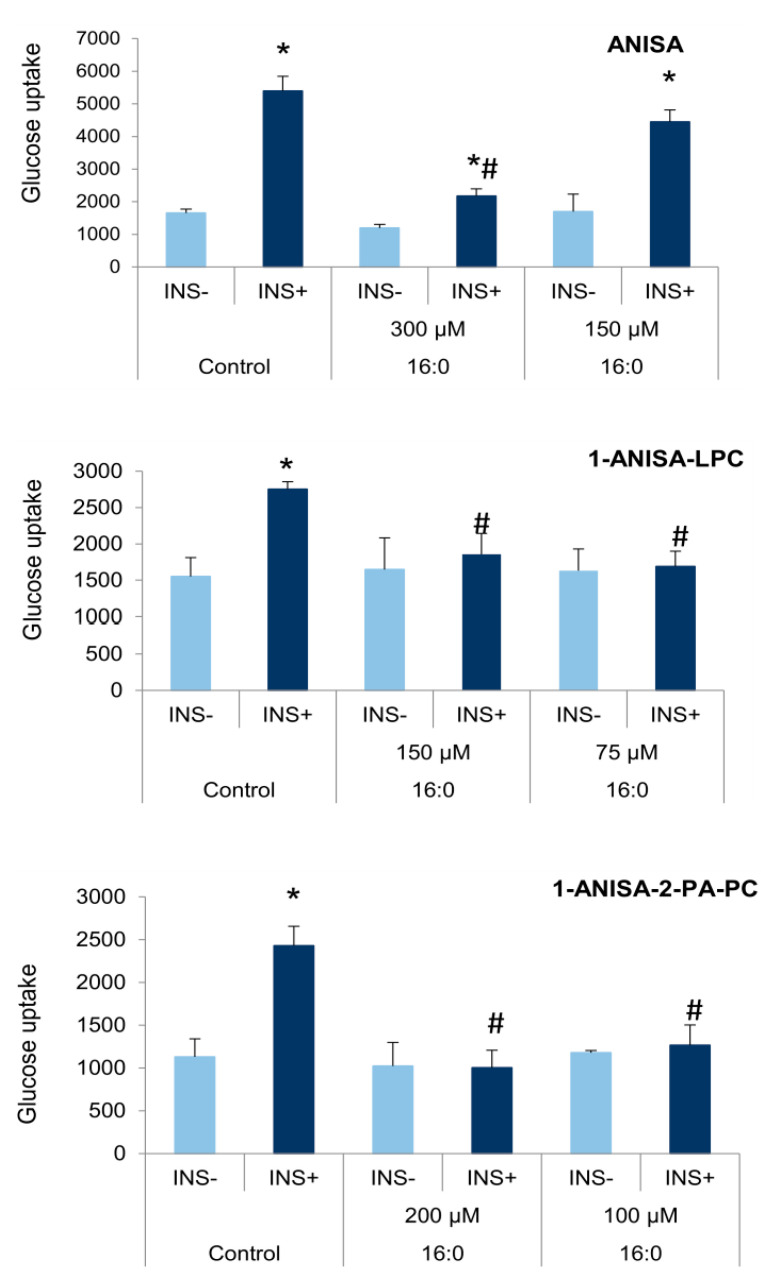
The insulin-stimulated glucose uptake after insulin resistance induction in 3T3-L1 adipocytes cultured with anisic acid and its derivatives. * statistical significance in relation to basal glucose uptake measured separately for each compound (INS−); # statistical significance between insulin-stimulated glucose uptake (INS+) in experimental and control cells; Student’s *t*-test, *p* < 0.05.

After insulin resistance induction by palmitic acid (16:0), the appropriate phospholipid derivatives were added to insulin-resistant cells and cultured for an additional period of 48 h. Anisic acid (ANISA) was added at the concentrations of 300 µM and 150 µM; 1-ANISA-LPC was used at the concentrations of 150 µM and 75 µM, and 1-ANISA-2-PA-PC was added to insulin-resistant adipocytes at the concentrations of 200 µM and 100 µM (Figure 5).

An increase in the insulin-stimulated glucose uptake in insulin-resistant adipocytes was observed in the cells treated with ANISA at both concentrations; glucose transport increased twice for the concentration of 300 µM (*p* = 0.002) and three times in the case of cell culture with 150 µM anisic acid (*p* = 0.008). The observed increase in glucose uptake by insulin-resistant adipocytes was lower than the one observed for control cells with normal insulin sensitivity. However, only at the concentration of 300 µM of ANISA did the insulin-stimulated glucose uptake differ between experimental and control cells and was statistically lower in insulin-resistant adipocytes treated with ANISA comparing to control adipocytes (*p* = 0.018).

On the other hand, the examined anisic acid derivatives did not stimulate glucose uptake in insulin-resistant cells. The insulin-stimulated glucose uptake was close to basic glucose uptake for two other tested compounds (1,2-diANISA-PC and 1-PA-2-ANISA-PC) (Figure 5). Moreover, the level of glucose uptake in insulin-resistant cells stimulated by insulin was significantly lower than in control cells with normal insulin sensitivity: 1 ANISA-LPC: 150 µM *p* = 0.018, 75 µM *p* = 0.013; 1-ANISA-2-PA-PC: 200 µM *p* = 0.006, 100 µM *p* = 0.007 (Figure 5).

### 3.4. The Influence of Cinnamic Acid, 3-Methoxycinnamic Acid, and Their Phospholipid Derivatives on Glucose Uptake in Insulin-Resistant Adipocytes

**Figure 6:**
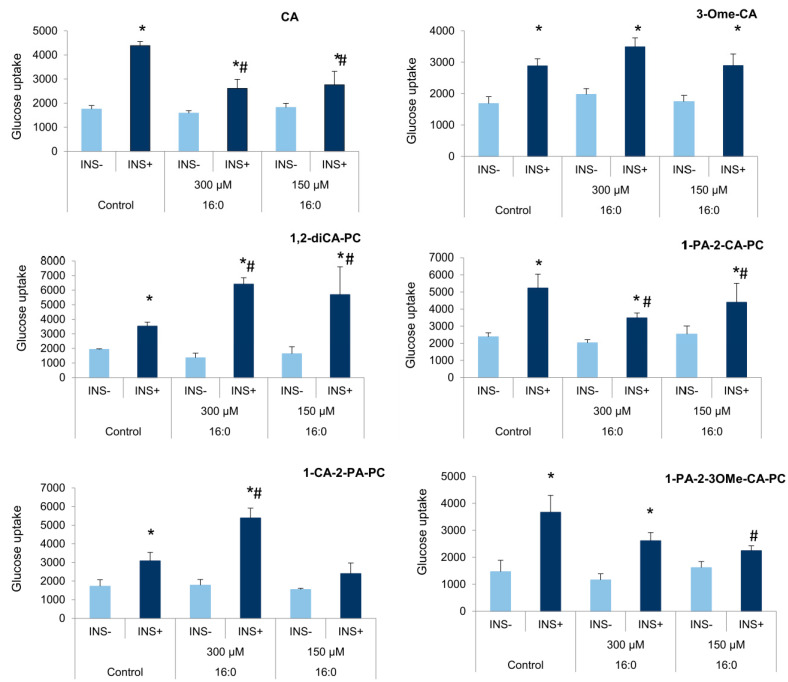
The insulin-stimulated glucose uptake after insulin resistance induction in 3T3-L1 adipocytes cultured with cinnamic acid, 3-methoxycinnamic acid, and its derivatives; * statistical significance in relation to basal glucose uptake measured separately for each compound (INS−); # statistical significance between insulin-stimulated glucose uptake (INS+) in experimental and control cells; Student’s *t*-test, *p* < 0.05.

Similarly, cinnamic acids (CA), 3-methoxycinnamic acid (3-OMe-CA), and their phospholipid derivatives were added to control and insulin-resistant cells for 48 h. We observed a significant increase in the insulin-stimulated glucose uptake for all analyzed compounds that are cinnamic acid and some of its derivatives. CA increased the insulin-stimulated glucose uptake in insulin-resistant adipocytes at experimental concentrations (300 µM *p* = 0.046 and 150 µM *p* = 0.033) comparing to basal glucose uptake (INS−). However, the level of glucose transport after insulin stimulation was still below the insulin-stimulated glucose uptake observed in control cells (300 µM *p* = 0.033, 150 µM *p* = 0.040, Figure 6).

The acid 3-OMe-CA increased the insulin-stimulated glucose uptake in insulin-resistant adipocytes at both tested concentrations (300 µM *p* = 0.012, 150 µM *p* = 0.017) comparing to basal glucose uptake rate, and the growth of insulin-stimulated glucose uptake in cells treated with 3-OMe-CA was similar to insulin-sensitive adipocytes (Figure 6).

Regarding 1,2-diCA-PC, we observed a significant increase in the insulin-stimulated glucose uptake in insulin-resistant adipocytes. At the concentration of 300 µM, the fourfold increase in the insulin-stimulated glucose uptake by insulin-resistant cells was observed (*p* < 0.000), while the lower concentration of 1,2-diCA-PC (150 µM) increased glucose absorption after insulin stimulation three times the value of basal glucose uptake (*p* = 0.023). Furthermore, the insulin-stimulated glucose uptake in adipocytes cultured with 1,2-diCA-PC was higher than in control adipocytes with proper insulin sensitivity (300 µM *p* < 0.000, 150 µM *p* = 0.034, Figure 6).

The phosphatidylcholine 1-PA-2-CA-PC also showed the properties of restoring proper insulin sensitivity, as it increased the insulin-stimulated glucose uptake in insulin-resistant adipocytes. A similar effect was observed for both tested concentrations of this compound, that is, 300 µM (*p* = 0.001) and 150 µM (*p* = 0.022). However, compared to the control adipocytes, the amount of glucose uptake after insulin stimulation was lower (300 µM *p* = 0.024, 150 µM *p* = 0.030, Figure 6), similar to that demonstrated by CA.

For the last two analyzed heterosubstituted phosphatidylcholines 1-CA-2-PA-PC and 1-PA-2-3-OMeCA-PC containing cinnamic acid and 3-methoxycinnamic acid in the position *sn*-1 and *sn*-2, respectively, the properties of overcoming insulin resistance were observed only for the higher concentration used in the study (300 µM). The substance 1-CA-2-PA-PC significantly increased the insulin-stimulated glucose uptake in insulin-resistant adipocytes; the increase was threefold compared to basal uptake (*p* = 0.014). On the other hand, at the concentration of 150 µM, only weak stimulation was observed. Moreover, the increase in glucose uptake after insulin stimulation in adipocytes treated with 300 µM 1-CA-2-PA-PC was higher than in control cells, properly responding to insulin (*p* = 0.013, Figure 6).

A similar effect was observed for 1-PA-2-3-OMe-CA-PC. We observed an increase in glucose uptake by insulin-resistant adipocytes only at a higher concentration of this compound (*p* = 0.007). No stimulation of glucose absorption was observed for the concentration of 150 µM. The level of the insulin-stimulated glucose uptake by IR adipocytes was slightly lower than in control cells (*p* = 0.059). On the other hand, the level of the insulin-stimulated glucose uptake by insulin-resistant adipocytes treated with 150 µM of 1-PA-2-3OMe-CA-PC was significantly lower than the one observed in cells with normal insulin sensitivity (*p* = 0.018, Figure 6), similar to basal glucose uptake.

## 4. Discussion

In the present study, we have shown that cinnamic acid and its derivatives obtained by adding phosphatidylcholine are highly effective in overcoming previously developed insulin resistance in 3T3-L1 adipocytes. Similarly, methyl cinnamate and its derivatives obtained by binding with phosphatidylcholine exhibited the properties of eliminating insulin resistance and restoring the correct cell response to insulin. Furthermore, the obtained results indicated that the effect of restoring insulin sensitivity was shown to be stronger in the derivatives containing two molecules of cinnamic acid in its structure (1,2-diCA-PC).

Previously, in the in vitro test performed on MIN6 cell line, it was shown that lysophosphatidylcholine with anisic acid is able to stimulate insulin secretion targeting G protein-coupled receptors [31]. Therefore, we decided to check if the methoxy derivative of phenolic acid in the form of conjugates with PC might also have a positive effect on the action of insulin in peripheral tissues. For this purpose, we selected three naturally occurring aromatic acids, such asanisic, cinnamic, and 3-methoxycinnamic acids. We evaluated them on the 3T3-L1 adipocytes, the most commonly studied cell model used as the adipose tissue model in cell culture to examine various types of metabolic disorders [33,34]. It responds to insulin similar to human adipocytes; as well, there are the same methods to induce resistance to insulin using the same chemical components [35,36]. Therefore, in the present study, we used palmitic acid to induce insulin resistance in mature 3T3-L1 adipocytes, which successfully developed resistance to insulin in experimental cells, proven by the insulin-stimulated glucose uptake measurements.

The derivatives of benzoic and cinnamic acid were selected based on literature data, which point to them as a class of widely distributed compounds that are considered important biologically active agents preventing the development of type 2 diabetes and its complications or other metabolic disorders such as obesity, lipids disorders, or insulin resistance [22,23].

Cinnamic acid and 3-methoxycinnamic acid are well-known naturally occurring aromatic acids that possess a potentially beneficial effect on the metabolism of adipocytes [23,26,37]. In our study, we analyzed the effect of these acids on insulin sensitivity in insulin-resistant 3T3-L1 adipocytes. We confirmed that among the studied acids, CA and 3-OMe-CA overcame the previously induced insulin resistance in 3T3-L1 adipocytes, which is consistent with other reports [23,26]. CA increased the insulin-stimulated glucose uptake in IR adipocytes. However, the increase was lower than in control cells, which suggests that CA increases insulin sensitivity but is unable to fully restore insulin sensitivity compared to control cells, regardless of the CA concentration. On the other hand, 3-OMe-CA fully restores insulin sensitivity, as the glucose uptake after insulin stimulation was comparable to control cells. A similar effect was observed for both concentrations used in the experimental study. For the higher concentration, the glucose uptake was even higher than in control adipocytes with proper insulin sensitivity; however, the differences were not statistically significant.

The beneficial effect of aromatic acids is limited in the in vivo models, mainly due to rapid metabolism after oral administration [38]. The other aspect of their limitation in oral administration is their bioavailability, as most phenolic acids are present in the bounded form and, thus, are poorly bioavailable [39]. For example, the content of bounded phenolic acids and their methoxy derivatives in barriers varies from 90% in raspberries, through 70% in strawberries to 30% in blueberries. In addition, low bioavailability is influenced by the rapid release of phenolic acids from the dietary matrix by intestinal bacteria as well as its rapid elimination by the urine or bile. One of the promising solutions to increase the bioavailability of phenolic acids and effective concentration, which increases their pro-health properties, might be their covalent binding with phospholipids. This was previously studied in our research groups and was proved in terms of anticancer properties [30]. It was previously shown that the conjugation with phospholipids is an effective method for increasing the biological properties of numerous natural compounds [31,40,41,42,43].

The present report shows that some selected aromatic acids after the conjugation with phosphatidylcholine are highly effective in overcoming previously developed insulin resistance in 3T3-L1 adipocytes compared to their free form and exhibit the ability to eliminate the insulin resistance and to restore the correct cell response to insulin. However, surprisingly, neither anisic acid nor its phospholipid derivatives showed a beneficial effect on insulin action in insulin-resistant adipocytes. Furthermore, although anisic acid increased the insulin-stimulated glucose uptake in IR adipocytes, the effect was relatively weak, comparing to control adipocytes with proper insulin sensitivity. Additionally, none of the examined anisic acid derivatives exhibited a beneficial effect on insulin action and insulin sensitivity in the examined IR adipocytes.

Very promising effects for the conjugates of PC with CA and 3-OMe-CA on the response to insulin of insulin-resistant adipocytes were observed. We have shown that the abovementioned derivatives allowed for overcoming the previously developed insulin resistance in 3T3-L1 adipocytes. Particularly noteworthy is the effect observed for 1,2 diCA-PC. The cinnamic acid derivative not only abolished the previously induced insulin resistance in 3T3-L1 adipocytes but also significantly increased the insulin-stimulated glucose uptake in insulin-resistant 3T3-L1 adipocytes, which significantly outstripped the response of the control cells. The insulin-stimulated glucose uptake was twice higher in adipocytes with the previously developed insulin resistance treated with 1,2-diCA-PC than in control adipocytes with proper insulin sensitivity. A similar effect was observed for both concentrations used in this study. This compound possesses two particles of cinnamic acids that increase its activity. On the other hand, this is not just an effect of a twice higher amount of CA. If it was just a matter of higher cinnamic acid molecules concentration, a similar effect between 150 µM of 1,2-diCA-PC and 300 µM cinnamic acid would be observed, but, indeed, cell responses differed from one another. It might be the effect of higher bioavailability or stability in the cell matrix. However, the conformational orientation of CA molecules also might be taken into account. We observed a similar effect in cells treated with 1-CA-2-PA-PC, that is, much higher insulin-stimulated glucose uptake in experimental adipocytes comparing to controls. Although the effect was observed only in the case of a higher concentration, the effect obtained exceeded the response of adipocytes with proper insulin sensitivity.

The results obtained indicate that the effect of restoring insulin sensitivity is strongly correlated with the structure of aromatic moiety and modified phospholipids. Among the tested compounds, the highest activity was observed for home substituted phosphatidylcholine (1,2-diCA-PC), containing two molecules of cinnamic acid in the skeleton of glycerol in both *sn*-1 and *sn*-2 positions. This type of derivative was more active than conjugates of PC with 3-methoxycinnamic acid. However, both of them were more effective than the derivative of anisic acid, which indicates that the acyclic unsaturated chain in the structure of phenylpropanoids is a crucial moiety for insulin sensitivity in insulin-resistant adipocytes.

There are numerous possible mechanisms of action by which the cinnamic acid and its derivatives might restore proper insulin action. It is not for sure a mechanism related to the insulin pathway, as we have not detected any changes in expression of insulin receptor gene (*INSR*) or glucose transporter gene (*SLC2A4*) in adipocytes cultured with examined compounds. Certainly, acyclic unsaturated chain in the structure of phenylpropanoids is crucial to induce the observed effect in the cell, thus, it might be the clue of the possible mechanism of action. It has been shown recently that unsaturated fatty acids stimulate the expression of the main transcription factor regulating the adipocytes metabolism along with insulin sensitivity, that is, PPARG [33]. Numerous other reports indicate that poly and monounsaturated fatty acids serve as a ligand for PPARG.

Additional attention should be paid to the low viability of adipocytes in compounds with the LPC group. Only one compound with the LPC group (1-ANISA-LPC) had a minor negative effect on cell survival. All LPC-bonded cinnamic acid and 3-methoxycinnamic acid derivatives significantly decreased the viability of cells even at relatively small concentrations.

## 5. Conclusions

To summarize, we have shown that both cinnamic acid and 3-methoxycinnamic acid have pro-health benefits in particular in restoring proper insulin sensitivity in insulin-resistant adipocytes. However, binding with phosphatidylcholine even increases the beneficial effect of these compounds. Thus, these compounds deserve particular interest for potential use in the treatment or prevention of type 2 diabetes.

## Data Availability

Data are available on request from the corresponding author.

## References

[1] Lebovitz H.E. (2001). Insulin Resistance: Definition and Consequences. Exp. Clin. Endocrinol. Diabetes.

[2] Abdul-Ghani M.A., DeFronzo R.A. (2010). Pathogenesis of Insulin Resistance in Skeletal Muscle. J. Biomed. Biotechnol..

[3] Ádány R., Pikó P., Fiatal S., Kósa Z., Sándor J., Bíró É., Kósa K., Paragh G., Bácsné Bába É., Veres-Balajti I. (2020). Prevalence of Insulin Resistance in the Hungarian General and Roma Populations as Defined by Using Data Generated in a Complex Health (Interview and Examination) Survey. Int. J. Environ. Res. Public Health.

[4] Rita S.L., Lubaki F.J.-P., Bompeka L.F., Ogunbanjo G.A., Ngwala L.P. (2019). Prevalence and Determinants of Psychological Insulin Resistance among Type 2 Diabetic Patients in Kinshasa, Democratic Republic of Congo. Afr. J. Prim Health Care Fam. Med..

[5] Sapunar J., Aguilar-Farías N., Navarro J., Araneda G., Chandia-Poblete D., Manríquez V., Brito R., Cerda A. (2018). High prevalence of overweight, obesity, insulin resistance and metabolic syndrome in rural children and adolescents. Rev. Med. Chil..

[6] Van der Aa M.P., Knibbe C.A.J., de Boer A., van der Vorst M.M.J. (2017). Definition of Insulin Resistance Affects Prevalence Rate in Pediatric Patients: A Systematic Review and Call for Consensus. J. Pediatr. Endocrinol. Metab..

[7] Brown A.E., Walker M. (2016). Genetics of Insulin Resistance and the Metabolic Syndrome. Curr. Cardiol. Rep..

[8] You D., Nilsson E., Tenen D.E., Lyubetskaya A., Lo J.C., Jiang R., Deng J., Dawes B.A., Vaag A., Ling C. (2017). Dnmt3a Is an Epigenetic Mediator of Adipose Insulin Resistance. Elife.

[9] Cierzniak A., Pawelka D., Kaliszewski K., Rudnicki J., Dobosz T., Malodobra-Mazur M. (2021). DNA Methylation in Adipocytes from Visceral and Subcutaneous Adipose Tissue Influences Insulin-Signaling Gene Expression in Obese Individuals. Int. J. Obes. Lond..

[10] Defronzo R.A. (1979). Glucose Intolerance and Aging: Evidence for Tissue Insensitivity to Insulin. Diabetes.

[11] Lankarani M., Valizadeh N., Heshmat R., Peimani M., Sohrabvand F. (2009). Evaluation of Insulin Resistance and Metabolic Syndrome in Patients with Polycystic Ovary Syndrome. Gynecol. Endocrinol..

[12] Bailey J.L., Zheng B., Hu Z., Price S.R., Mitch W.E. (2006). Chronic Kidney Disease Causes Defects in Signaling through the Insulin Receptor Substrate/Phosphatidylinositol 3-Kinase/Akt Pathway: Implications for Muscle Atrophy. J. Am. Soc. Nephrol..

[13] Swan J.W., Anker S.D., Walton C., Godsland I.F., Clark A.L., Leyva F., Stevenson J.C., Coats A.J. (1997). Insulin Resistance in Chronic Heart Failure: Relation to Severity and Etiology of Heart Failure. J. Am. Coll. Cardiol..

[14] Pagano G., Cavallo-Perin P., Cassader M., Bruno A., Ozzello A., Masciola P., Dall’omo A.M., Imbimbo B. (1983). An in Vivo and in Vitro Study of the Mechanism of Prednisone-Induced Insulin Resistance in Healthy Subjects. J. Clin. Investig..

[15] Hruz P.W. (2008). HIV Protease Inhibitors and Insulin Resistance: Lessons from in-Vitro, Rodent and Healthy Human Volunteer Models. Curr. Opin. HIV AIDS.

[16] Banach M., Malodobra-Mazur M., Gluba A., Katsiki N., Rysz J., Dobrzyn A. (2013). Statin Therapy and New-Onset Diabetes: Molecular Mechanisms and Clinical Relevance. Curr. Pharm. Des..

[17] Kim J.K. (2009). Hyperinsulinemic-Euglycemic Clamp to Assess Insulin Sensitivity In Vivo. Methods Mol. Biol..

[18] Association A.D. (2020). Standards of Medical Care in Diabetes—2020 Abridged for Primary Care Providers. Clin. Diabetes.

[19] Bailey C.J. (2017). Metformin: Historical Overview. Diabetologia.

[20] Hong J., Zhang Y., Lai S., Lv A., Su Q., Dong Y., Zhou Z., Tang W., Zhao J., Cui L. (2013). Effects of Metformin versus Glipizide on Cardiovascular Outcomes in Patients with Type 2 Diabetes and Coronary Artery Disease. Diabetes Care.

[21] Rochlani Y., Pothineni N.V., Kovelamudi S., Mehta J.L. (2017). Metabolic Syndrome: Pathophysiology, Management, and Modulation by Natural Compounds. Therap. Adv. Cardiovasc. Disease.

[22] Belwal T., Nabavi S.F., Nabavi S.M., Habtemariam S. (2017). Dietary Anthocyanins and Insulin Resistance: When Food Becomes a Medicine. Nutrients.

[23] Hafizur R.M., Hameed A., Shukrana M., Raza S.A., Chishti S., Kabir N., Siddiqui R.A. (2015). Cinnamic Acid Exerts Anti-Diabetic Activity by Improving Glucose Tolerance in Vivo and by Stimulating Insulin Secretion in Vitro. Phytomedicine.

[24] Czarnecka M., Świtalska M., Wietrzyk J., Maciejewska G., Gliszczyńska A. (2018). Synthesis, Characterization, and In Vitro Cancer Cell Growth Inhibition Evaluation of Novel Phosphatidylcholines with Anisic and Veratric Acids. Molecules.

[25] Hsu F.L., Chen Y.C., Cheng J.T. (2000). Caffeic Acid as Active Principle from the Fruit of Xanthium Strumarium to Lower Plasma Glucose in Diabetic Rats. Planta Med..

[26] Adisakwattana S., Roengsamran S., Hsu W.H., Yibchok-anun S. (2005). Mechanisms of Antihyperglycemic Effect of P-Methoxycinnamic Acid in Normal and Streptozotocin-Induced Diabetic Rats. Life Sci..

[27] Yibchok-anun S., Adisakwattana S., Moonsan P., Hsu W.H. (2008). Insulin-Secretagogue Activity of p-Methoxycinnamic Acid in Rats, Perfused Rat Pancreas and Pancreatic Beta-Cell Line. Basic Clin. Pharmacol. Toxicol..

[28] Insulin-Releasing Properties of a Series of Cinnamic Acid Derivatives In Vitro and In Vivo-PubMed. https://pubmed.ncbi.nlm.nih.gov/18651742/.

[29] Adisakwattana S., Hsu W.H., Yibchok-anun S. (2011). Mechanisms of P-Methoxycinnamic Acid-Induced Increase in Insulin Secretion. Horm. Metab. Res..

[30] Czarnecka M., Świtalska M., Wietrzyk J., Maciejewska G., Gliszczyńska A. (2018). Synthesis and Biological Evaluation of Phosphatidylcholines with Cinnamic and 3-Methoxycinnamic Acids with Potent Antiproliferative Activity. RSC Adv..

[31] Drzazga A., Okulus M., Rychlicka M., Biegała Ł., Gliszczyńska A., Gendaszewska-Darmach E. (2020). Lysophosphatidylcholine Containing Anisic Acid Is Able to Stimulate Insulin Secretion Targeting G Protein Coupled Receptors. Nutrients.

[32] Małodobra-Mazur M., Cierzniak A., Kaliszewski K., Dobosz T. (2021). PPARG Hypermethylation as the First Epigenetic Modification in Newly Onset Insulin Resistance in Human Adipocytes. Genes.

[33] Malodobra-Mazur M., Cierzniak A., Dobosz T. (2019). Oleic Acid Influences the Adipogenesis of 3T3-L1 Cells via DNA Methylation and May Predispose to Obesity and Obesity-Related Disorders. Lipids Health Dis..

[34] Dordevic A.L., Konstantopoulos N., Cameron-Smith D. (2014). 3T3-L1 Preadipocytes Exhibit Heightened Monocyte-Chemoattractant Protein-1 Response to Acute Fatty Acid Exposure. PLoS ONE.

[35] Shinjo S., Jiang S., Nameta M., Suzuki T., Kanai M., Nomura Y., Goda N. (2017). Disruption of the Mitochondria-Associated ER Membrane (MAM) Plays a Central Role in Palmitic Acid-Induced Insulin Resistance. Exp. Cell Res..

[36] Pinel A., Rigaudière J.-P., Jouve C., Capel F. (2018). Modulation of Insulin Resistance and the Adipocyte-Skeletal Muscle Cell Cross-Talk by LCn-3PUFA. Int. J. Mol. Sci..

[37] Scazzocchio B., Varì R., Filesi C., D’Archivio M., Santangelo C., Giovannini C., Iacovelli A., Silecchia G., Li Volti G., Galvano F. (2011). Cyanidin-3-O-β-Glucoside and Protocatechuic Acid Exert Insulin-like Effects by Upregulating PPARγ Activity in Human Omental Adipocytes. Diabetes.

[38] Bento-Silva A., Koistinen V.M., Mena P., Bronze M.R., Hanhineva K., Sahlstrøm S., Kitrytė V., Moco S., Aura A.-M. (2020). Factors Affecting Intake, Metabolism and Health Benefits of Phenolic Acids: Do We Understand Individual Variability?. Eur. J. Nutr..

[39] Hole A.S., Rud I., Grimmer S., Sigl S., Narvhus J., Sahlstrøm S. (2012). Improved Bioavailability of Dietary Phenolic Acids in Whole Grain Barley and Oat Groat Following Fermentation with Probiotic Lactobacillus Acidophilus, Lactobacillus Johnsonii, and Lactobacillus Reuteri. J. Agric. Food Chem..

[40] Gliszczyńska A., Niezgoda N., Gładkowski W., Czarnecka M., Świtalska M., Wietrzyk J. (2016). Synthesis and Biological Evaluation of Novel Phosphatidylcholine Analogues Containing Monoterpene Acids as Potent Antiproliferative Agents. PLoS ONE.

[41] Gliszczyńska A., Niezgoda N., Gładkowski W., Świtalska M., Wietrzyk J. (2017). Isoprenoid-Phospholipid Conjugates as Potential Therapeutic Agents: Synthesis, Characterization and Antiproliferative Studies. PLoS ONE.

[42] Drzazga A., Kamińska D., Gliszczyńska A., Gendaszewska-Darmach E. (2021). Isoprenoid Derivatives of Lysophosphatidylcholines Enhance Insulin and GLP-1 Secretion through Lipid-Binding GPCRs. Int. J. Mol. Sci..

[43] Palko-Łabuz A., Gliszczyńska A., Skonieczna M., Poła A., Wesołowska O., Środa-Pomianek K. (2021). Conjugation with Phospholipids as a Modification Increasing Anticancer Activity of Phenolic Acids in Metastatic Melanoma-In Vitro and In Silico Studies. Int. J. Mol. Sci..

